# Risk Factors for Predicting Osteoporosis in Patients Who Receive Thyrotropin Suppressive Levothyroxine Treatment for Differentiated Thyroid Carcinoma

**DOI:** 10.4274/mirt.galenos.2019.89410

**Published:** 2019-06-24

**Authors:** Çiğdem Soydal, Elgin Özkan, Demet Nak, Atilla Halil Elhan, Nuriye Özlem Küçük, Metin Kemal Kır

**Affiliations:** 1 Ankara University Faculty of Medicine, Department of Nuclear Medicine, Ankara, Turkey; 2 Ankara University Faculty of Medicine, Department of Biostatistics, Ankara, Turkey

**Keywords:** Differentiated thyroid carcinoma, osteoporosis, thyroid-stimulating hormone suppression treatment

## Abstract

**Objectives:**

Endogenous hyperthyroidism accelerates bone *turnover* and shortens the normal bone *remodeling* cycle, which results in reduced bone density. It is estimated that suppressive levothyroxine (LT4) therapy also decreases bone density. The aim of this study was to define risk factors for osteoporosis development in patients under thyrotropin-stimulating hormone (TSH) suppressive treatment for differentiated thyroid cancer (DTC).

**Methods:**

Patients with a diagnosis of low or intermediate risk group DTC according to the American Thyroid Association 2015 guidelines and who have been receiving LT4 suppression therapy and were physically fit to undergo femur and lumbar vertebra bone density study were included in the study. Patients lacking information on demographic data, medical history, preoperative thyroid hormone status, or routine follow-up data were excluded from the study. A study form consisting of patient information on possible risk factors for osteoporosis such as gender, age, menopausal status, smoking, family history of osteoporosis, preoperative thyroid hormone status, postoperative hypoparathyroidism history, mean serum TSH levels, and duration of TSH suppression was created and filled out for each participant. Bone mineral densitometries of the femur and lumbar vertebrae were measured along with serum vitamin D and parathyroid hormone levels.

**Results:**

During TSH suppression (mean 7.2±4.5 years, range: 1-26), osteoporosis was detected in 89 (9.6%) patients. The mean time to develop osteoporosis was significantly different in patients with or without a family history of osteoporosis (15.3±0.4 versus 20.3±0.6 years; p=0.002). Similarly, the mean time to develop osteoporosis for was found to be significantly shorter in postmenopausal patients than that for premenopausal women (18.6±0.7 versus 20.4±0.4 years; p<0.001). Male gender (p<0.001), a family history of osteoporosis (p=0.001) and menopausal state (p<0.001) were identified as independent predictive factors for developing osteoporosis.

**Conclusion:**

Postmenopausal women, men, and patients with a family history who receive TSH-suppression treatment have a tendency to develop osteoporosis.

## Abstract (tr)

**Amaç:**

Endojen hipertiroidi kemik *turn-overini* hızlandırır ve normal kemik *remodeling* döngüsünü kısaltır, bu da azalmış kemik yoğunluğuyla sonuçlanır. Süpresif levotiroksin (LT4) tedavisinin de kemik yoğunluğunu azalttığı düşünülmektedir. Bu çalışmada, diferansiye tiroid kanseri (DTK) için tirotropin-stimüle edici hormon (TSH) süpresif tedavi altındaki hastalarda osteoporoz gelişimi için risk faktörlerinin tanımlanması amaçlanmıştır.

**Yöntem:**

Amerikan Tiroid Derneği 2015 rehberine göre düşük veya orta riskli DTK tanısı ile takipli, LT4 süpresyon tedavisi alan; bazal tedavi sonrası rutin klinik takip bilgilerine ulaşılan; hasta dahil etme formu doldurmak için gerekli ve yeterli demografik ve medikal takip bilgileri bulunan; genel durumu femur ve lumbal vertebra kemik dansitometri çalışması yapılması için uygun olan hastalar çalışmaya dahil edildi. Osteoporoz için olası risk faktörleri olan cinsiyet, yaş, menopoz durumu, sigara, ailede osteoporoz hikayesi, preoperatif tiroid hormon durumu, postoperatif hipoparatiroidi hikayesi, ortalama serum TSH düzeyi ve TSH süpresyon süresini içeren bir hasta dahil etme formu dolduruldu. Dahil edilen hastalara bu aşamada femur boynu ve lumbal vertebralardan kemik mineral dansitometrisi yapıldı, serum D vitamin ve parathormon düzeylerine bakıldı.

**Bulgular:**

Ortalama TSH süpresyon süresi 7,2±4,5 (aralık: 1-26) yıl idi, hastaların 89’unda (%9,6) osteoporoz saptandı. Postmenopozal olgularda osteoporoz gelişmesine kadar geçen ortalama süre premenopozal olgulardan anlamlı şekilde kısaydı (18,6±0,7’ye karşın 20,4±0,4 yıl; p<0,001). Erkek cinsiyet (p<0,001), aile hikayesi (p=0,001) ve menopoz durumu (p<0,001) osteoporoz gelişimi için bağımsız risk faktörleri olarak saptandı.

**Sonuç:**

TSH süpresyon tedavisi alan postmenopozal kadınlar, erkek ve aile öyküsü olan hastalar osteoporoz gelişimine eğilim göstermektedir.

## Introduction

Differentiated thyroid carcinoma (DTC) is the most common endocrine neoplasia. Although the incidence of DTC is increasing its mortality rate remains stable (1,2). After initial treatment with a thyroidectomy with/without radioiodine treatment, patients are treated with levothyroxine (LT4) therapy to suppress thyrotropin-stimulating hormone (TSH) since suppression of serum TSH levels reduces tumor recurrence rates (3).

Endogenous hyperthyroidism has been shown to reduce bone density because hyperthyroidism accelerates bone turnover and shortens the normal bone remodeling cycle (4).For this reason, suppression LT4 therapy might cause a decrease in bone density. Considering the long life expectancy for DTC patients, treatment related comorbidities could affect their quality of life.

Although several studies have been designed to explore the correlation between bone density changes and LT4 treatment, conflicting findings have been reported (4,5). Most of the reported studies have included small number of patients, and despite this limitation bone density seems to decrease in at least some DTC patients. In this large series, we analyzed DTC patients’ bone density after considering several demographic features, comorbidities, and treatment-related risk factors. In our analysis, we aimed to define additional risk factors for developing osteoporosis in patients who received TSH-suppressive LT4 treatment for DTC.

## Materials and Methods

### Patients

This study included prospective and retrospective components and was approved by the Institutional Ethical Committee of Ankara University Medical Faculty (approval number: 11-489-16). After receiving informed consent for the prospective component, patient inclusion was continued for the period between June 2016 and Jan 2018. All patients had received radioiodine treatment for DTC in Ankara University Medical Faculty Department of Nuclear Medicine. Patient inclusion criteria were based on several parameters: (1) low or intermediate risk group DTC diagnosis according to the American Thyroid Association (ATA) 2015 guidelines; (2) receiving LT4 suppression therapy after initial treatment; (3) available routine follow-up data after initial therapy; (4) known preoperative thyroid hormone status; (5) sufficient and available demographic and medical history data to fill-in the study form; and (6) be physically fit enough to undergo a femur and lumbar vertebra bone density study (6).

### Data Generation

A study form including information on possible risk factors for osteoporosis such as gender, age, menopausal status, smoking, family history of osteoporosis, preoperative thyroid hormone status, postoperative hypoparathyroidism history, mean serum TSH levels, and duration of TSH suppression was created and filled out. Mean serum TSH levels were calculated from at least two serum TSH measurements per year, excluding endogenous or exogenous short duration stimulated TSH levels. The duration of TSH suppression was calculated as the interval between TSH-suppression LT4 treatment initiation and the date of patient inclusion. After selection of patients, bone mineral densitometries of the femur and lumbar vertebrae, serum vitamin D, and parathyroid hormone measurements were performed. T and Z scores of the femur and lumbar vertebrae were used for analysis. The presence of osteoporosis was accepted as T scores <-2.5. In patients who already had osteoporosis at the time of study initiation, the date of osteoporosis diagnosis was retrospectively obtained from patient files.

### Statistical Analysis

The differences in proportions between groups were compared by using chi-square test. The survival estimations were performed using the method of Kaplan-Meier algorithm, and the comparison between groups was evaluated with the log-rank test. Multiple Cox regression proportional hazard model was used to determine independent predictors of osteoporosis development (7). p value less than 0.05 was considered as significant. SPSS version 20.0 (IBM, Chicago, Illinois, USA) was used for statistical analyses.

## Results

### Patients

A total of 929 patients (813 female, 116 male, mean age: 52.33±7.2) who received TSH suppression therapy for DTC were included. Patient descriptive data are presented in Table 1.

### Risk Factors to Development of Osteoporosis

During TSH suppression (mean 7.2±4.5 years, range: 1-26), osteoporosis was detected in 89 (9.6%) patients. The rate of osteoporosis in patients with and without a family history of osteoporosis was 13% and 8%, respectively. Osteoporosis detection rates were calculated as 0.6%, 15%, and 12% in premenopausal and postmenopausal women and men, respectively. Preoperative hyperthyroidism was found to be significantly correlated with the presence of osteoporosis based on chi-squared analysis with 15% versus 8% (p=0.003); however, this significance was lost in multivariate Cox regression analysis. Although mean serum TSH levels were not significant factors for the presence of osteoporosis, osteoporosis detection rates seem to decrease in patients with TSH levels >0.4 mIU/L. Osteoporosis rates for different risk groups are summarized in Table 2.

**Figure 1:**
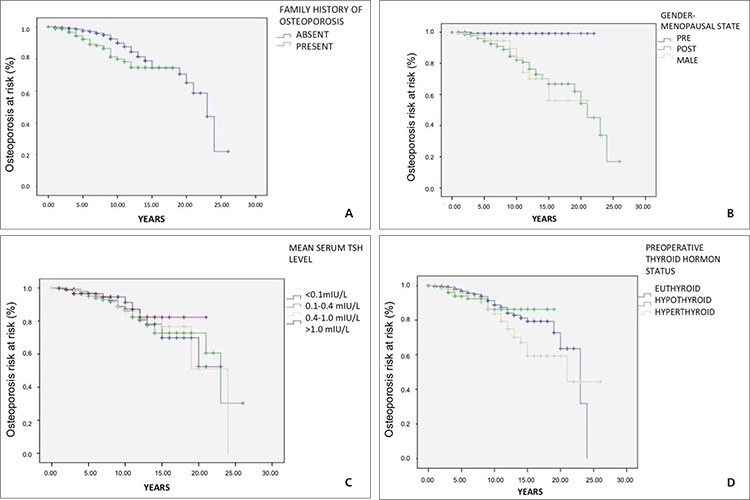
A, B, C, D) Kaplan-Meier curve for developing osteoporosis according to different risk groups TSH: Thyrotropin-stimulating hormone

The mean time to develop osteoporosis for patients with and without a family history of osteoporosis was significantly different (15.3±0.4 versus 20.3±0.6 years; p=0.002). Similarly, the mean time to develop osteoporosis was found to be significantly shorter in postmenopausal patients as compared to premenopausal women (18.6±0.7 versus 20.4±0.4 years; p<0.001). The mean time to develop osteoporosis according to patient characteristics is shown in Table 3. The mean time to develop osteoporosis among patient groups according to risk factors was not significant in Kaplan-Meier analysis (p>0.05). Kaplan-Meier curves for different groups are presented in Figures 1A, 1B, 1C, 1D.

Using the Cox proportional hazards regression analysis, male gender [hazard ratio (HR): 20.510, 95% confidence interval (CI): 4.644-90.579, p<0.001], family history of osteoporosis (HR: 2.215, 95% CI: 1.365-3.308, p=0.001) and menopausal state (post menopausal: HR: 18.488, 4.534-75.389, p<0.001; HR: 20.510, 4.644-90.579, p<0.001) were identified as independent predictive factors for developing osteoporosis (Table 4). According to multiple Cox regression proportional hazard analysis, other risk factors were not found to be significant (p>0.05).

## Discussion

It is considered that remnant DTC cells behave in a manner similar to benign thyrocytes from which they originated. TSH stimulates the number, size and activity of thyrocytes (8). The rationale for this approach was based on observations that the incidence of thyroid cancer is correlated with serum TSH levels in the normal population (9). Hence, the primary aim of TSH suppression therapy is to lower endogenous TSH levels to reduce the risk of disease recurrence.

In the literature, different outcomes have been reported concerning the benefits of long-term TSH suppression in DTC patients. Lower serum TSH levels have been shown to be an independent predictor for disease progression in patients with a high risk of tumor recurrence. Interestingly, a similar effect has not been demonstrated on patients with stage 1 or 2 disease (10). Moreover, a meta-analysis including 10 studies did not demonstrate any benefits from TSH suppression (11). The National Thyroid Cancer Cooperative Study Group Registry published a study including 1548 patients. In contrast to our study, in their analysis, TSH suppression improved overall survival in stage 2 patients (12).Similarly, Hovens et al. (13) have reported results of 366 patients treated with total thyroidectomy followed by radioiodine treatment. They found that serum TSH levels >4.5 mU/L was an independent predictor for death, and TSH levels >2 mU/L were also associated with DTC-related deaths and recurrence in patients with T1-3, M0 tumors. Also, Pujol et al. (14) reported that suppressed serum TSH levels were associated with an increase in relapse-free survival in patients with DTC.

Controversial results based on these analyses have led to discussions about optimal TSH level and duration of suppression for low-intermediate risk group patients with respect to therapy-related side effects. Thyroid hormones act directly on the skeleton, and endogenous hyperthyroidism is known to be related with a high risk of osteoporosis (15). Known risks of iatrogenic overt or subclinical hyperthyroidism are osteoporosis, osteopenia, and/or atrial fibrillation. For this reason, slightly subnormal or normal TSH levels are recommended for long term periods (16).Two cohort studies have demonstrated that postmenopausal DTC patients with fully suppressed TSH levels have a high risk of osteoporosis (17,18).In our study, we aimed to analyze additional risk factors for developing osteoporosis in a large cohort. A family history of osteoporosis and menopausal status were found to be significant factors favoring osteoporosis development. The presence of preoperative hyperthyroidism could also be another risk factor. Interestingly, we could not find any significant correlation between mean serum TSH levels and presence of osteoporosis. However, osteoporosis detection rates tend to decrease in patients with mean TSH levels >0.4 mIU/L.

Another interesting finding of our analysis was that male patients who received TSH suppression therapy were found to have a 20-fold increase in developing osteoporosis as compared to premenopausal women. Most studies have included only female patients for osteoporosis analysis. Reverter et al. (19) analyzed bone mineral densities and bone fractures in male patients receiving long-term TSH suppressive therapy. They compared bone mineral density and bone turnover parameters from 33 DTC patients with age- and body mass index-matched control groups. They did not find any significant differences between bone turnover parameters, including the T and Z scores, between groups.We could not compare our study parameters with an age-matched group. However, the number of included male patients in this study was higher than that in the previous study. A total of 66 patients and 67 controls were included in a recent meta-analysis on the effects of TSH suppression in men. The authors did not find any significant correlation between TSH suppression and lower BMD values in men (20). For this reason, the osteoporosis rate in male patients receiving TSH suppression therapy needs further clarification with prospective randomized control studies.

The ATA 2015 guidelines recommend 0.1-0.5 mU/L levels as an initial TSH goal for low-risk group patients with indeterminate or incomplete response as well as for intermediate risk group patients based on data on this subject. The guide recommends continuation therapy with 0.5-2.0 mU/L levels for low and intermediate risk group patients with excellent response. It is reasonable for clinicians to consider disease stage, response to initial treatment, and personal risk factors to develop osteoporosis in order to personalize a patient’s TSH suppression therapy. The risk of disease recurrence and TSH suppression-related risks should be balanced. Postmenopausal women, men, and patients with a family history of osteoporosis have a high rate of osteoporosis under TSH-suppression LT4 treatment. Preoperative hyperthyroidism and mean serum TSH levels seem to be possible predictors of developing osteoporosis, although not statistically significant.

## Conclusion

The current data suggest that personalized TSH suppression treatment, based on DTC risk group and patient-related risk factors to develop osteoporosis, might be beneficial.

Postmenopausal women, men, and patients with a family history who are under TSH-suppression treatment have a high rate of osteoporosis. Thus, male or postmenopausal female patients with low/intermediate risk DTC and a family history of osteoporosis should be closely followed-up.

## Figures and Tables

**Table 1:**
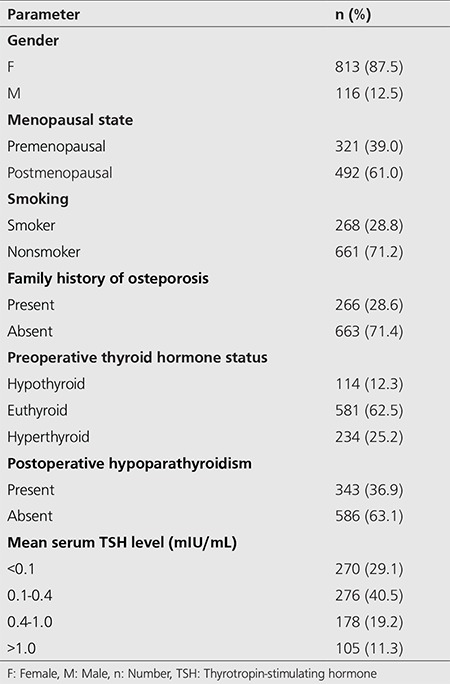
Descriptive data of patients

**Table 2:**
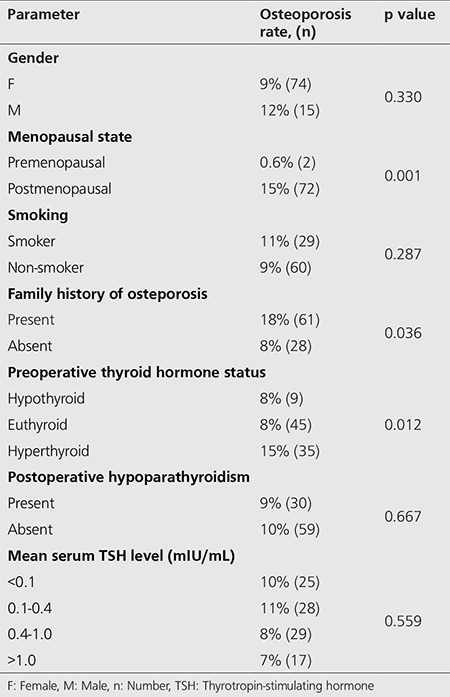
Osteoporosis rates of different groups

**Table 3:**
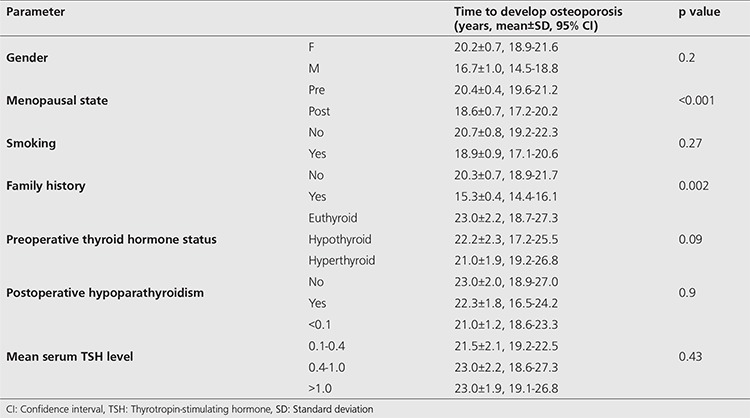
The mean time to develop osteoporosis according to patient characteristics

**Table 4:**
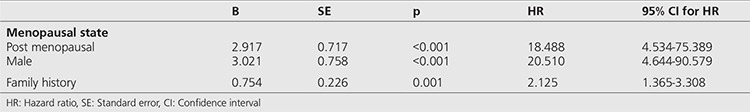
Independent predictive factors of developing osteoporosis
